# Hemodynamic Instability in Emergency Department: Giant Rectus Sheath Hematoma and Point-of-Care Ultrasonography

**DOI:** 10.7759/cureus.13669

**Published:** 2021-03-03

**Authors:** Yagmur Ay, Mustafa Emin Canakci, Tuncer Temel, Engin Ozakin, Nurdan Acar

**Affiliations:** 1 Emergency Medicine, Eskisehir Osmangazi University, Eskisehir, TUR; 2 Gastroenterology and Hepatology, Eskisehir Osmangazi University, Eskisehir, TUR

**Keywords:** rectus sheath hematoma, paracentesis, ultrasonography, emergency department

## Abstract

Iatrogenic rectus sheath hematoma (RSH) developed after paracentesis is a rare but life-threatening complication. Mortality rates of patients may increase due to delays in treatment and comorbid conditions. In this article, we present the case of a patient who was unstable in the emergency department and was diagnosed with RSH using point-of-care ultrasonography (POCUS). The importance of POCUS has increased as hematoma manifestations of patients with severe ascites tend to be obscured. POCUS has varied uses in the emergency department, and in this article we emphasize the use of POCUS in a life-threatening case of RSH.

## Introduction

Rectus sheath hematoma (RSH) occurs as a result of accumulation of blood in the rectus sheath due to rupture of the epigastric vessels or tear in the muscle fibers. RSH is a rare presentation in the emergency department (ED). In particular, iatrogenic RSH secondary to paracentesis has been reported in less than 1% of the cases [1,2]. Clinically significant and life-threatening hemorrhagic complications are observed much more rarely [1]. Although RSH is a self-limiting condition and spontaneous recovery is often observed, mortality is reported in patients with severe coagulopathy [3].

The use of point-of-care ultrasound (POCUS) in the ED is increasing every day. RSH can be diagnosed promptly with POCUS [4]. Thus, the use of POCUS as a rapid, noninvasive, and inexpensive method of differential diagnosis of critical patients will continue to increase. In this case report, the diagnosis of iatrogenic RSH was made by POCUS in the ED and treatment was initiated.

## Case presentation

A 65-year-old female patient was brought to the ED with a complaint of abdominal pain. The patient had a known history of liver cirrhosis and had undergone paracentesis three days ago due to widespread ascites. She had essential thrombocytosis, rheumatoid arthritis, and hypertension. Vital signs included blood pressure of 75/45 mmHg, heart rate 155/minutes, oxygen saturation 95%, and body temperature 36.3°C. There was tenderness in the abdomen and bulging flanks with fluid wave. There was no ecchymosis, warmth, or discharge at the site of paracentesis. The rest of the examination revealed no distinctive findings except for tachycardia.

Initial laboratory results were as follows: hemoglobin of 8.1 g/dL, leukocyte 23,020/uL, platelet 941,000/uL, pH 7.375, lactate 6.3 mmol/L, HCO_3_ 18.9 mmol/L, and international normalized ratio (INR) of 1.39.

**Figure 1:**
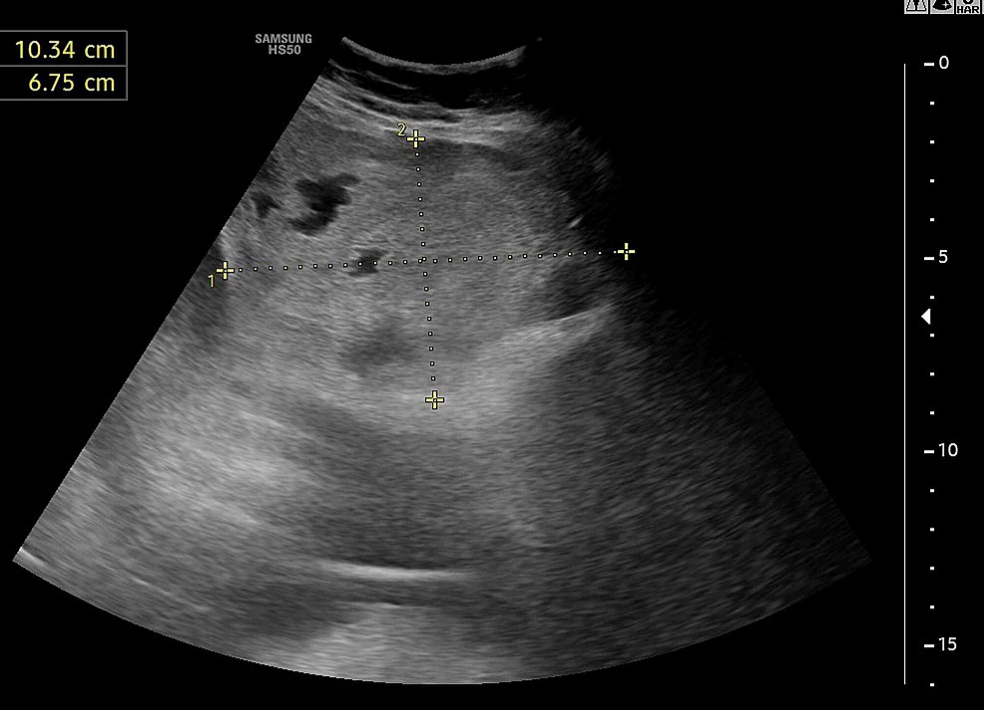
POCUS examination of the paracentesis site. POCUS, point-of-care ultrasonography

Because the patient was intervened three days ago, septic shock due to peritonitis was suspected. POCUS was performed for differential diagnosis, and a hyperdense area with anechoic areas within a diameter of about 11 cm above the intervention site in the abdomen was suspected as hematoma. Power Doppler examination revealed no signs of active bleeding and no pseudoaneurysm (Figure 1, Video 1). Vital parameters of the patient were unstable, and transfusion was initiated due to a concern of stage 4 hemorrhagic shock. The patient was emergently intubated following deterioration in her level of consciousness.

**Video 1:** Ultrasonographic evaluation of the abdomen. Giant rectus hematoma without Doppler flow in color mode.

**Figure 2:**
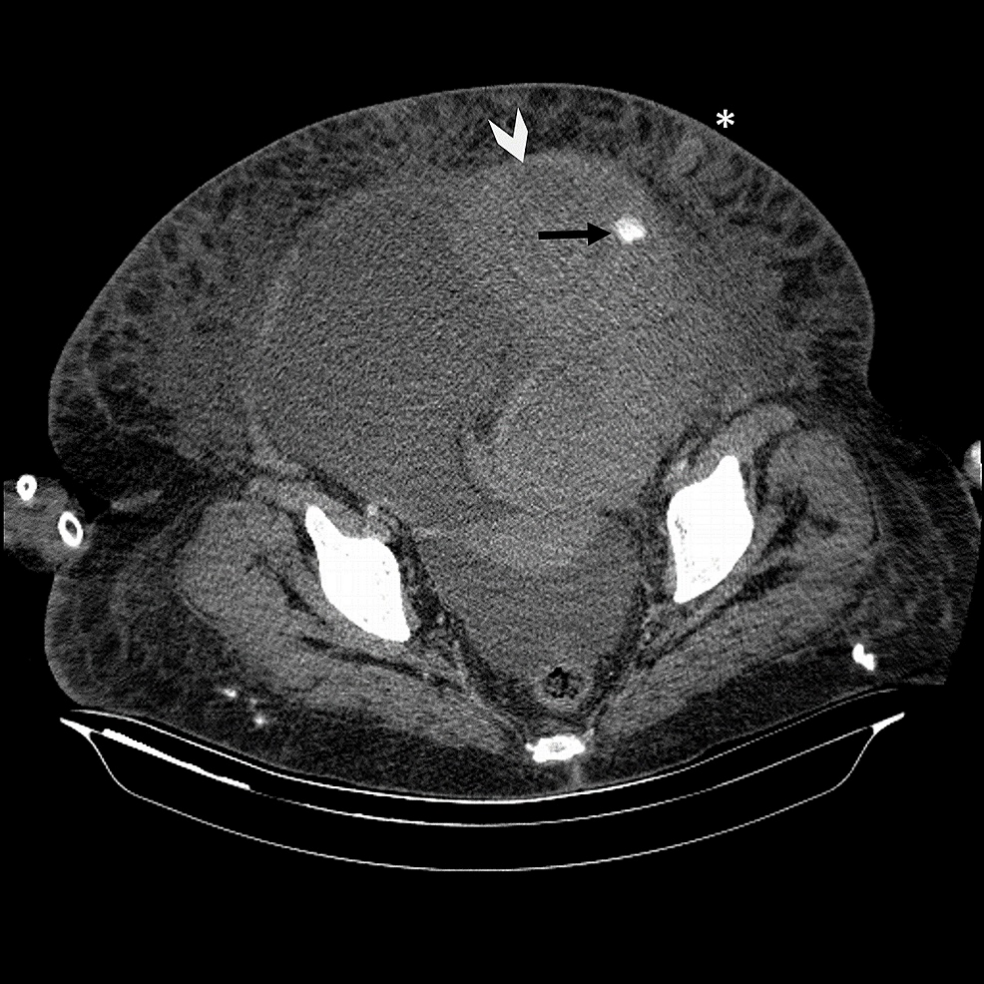
CT findings of rectus hematoma. Black arrow: active contrast leak in the inferior epigastric artery area. Arrowhead: giant hematoma. Asteriks: paracentesis site. CT, computed tomography

Upon erythrocyte suspension transfusion, the patient’s tachycardia improved and blood pressure was 90/60 mmHg. RSH with diffuse ascites and 11 × 11 cm active extravasation and contrast transition was observed on abdominal computed tomography, which was performed for determining the area of hemorrhage (Figure 2). Patient had active bleeding from the inferior epigastric artery; therefore, percutaneous intervention was not considered by Interventional Radiology.

The patient was admitted to the intensive care and replacement therapy was continued. However, the patient expired due to cardiac arrest which occurred 48 hours after admission.

## Discussion

Although RSH is a rare presentation in the ED, it should be considered in critically ill patients. It particularly occurs in patients who sustain abdominal trauma and undergo interventional operations, as well as those who are on anticoagulants [5]. RSH is often spontaneously reabsorbed and is a self-limiting condition; however, very rarely, it can lead to life-threatening hemorrhages. Long-term follow-up of patients with coagulopathy, especially patients undergoing paracentesis for liver cirrhosis, and emergency hospital admission reduces mortality and morbidity.

In previous case reports related to RSH, the area of hematoma was evident on physical examination; however, the distinction was not clear in our case because of the prominent abdominal distension [4]. With the use of POCUS, we were able to confirm the diagnosis of RSH and rule out the presence of abscess collection or peritonitis. Resuscitation of several critical patients is performed appropriately by following the POCUS protocols, but its out-of-protocol use still needs to be carefully evaluated. Echocardiography, pulmonary evaluation, abdominal evaluation, and aortic evaluation are performed according to the protocols. Furthermore, POCUS should be performed to explain the patient’s hypotension, as was the case presented here.

RSH should be considered as an important differential diagnosis in patients with abdominal pain presenting to the ED, and a history of coagulopathy should be questioned for hemorrhagic complications. In the case presented in this report, although the patient had mild coagulopathy, it was believed that the hemorrhagic tendency was due to thrombocytosis. Although thrombotic risk is greater than hemorrhagic risk in patients with thrombocytosis, the incidence of hemorrhagic complications is observed to be around 5% [6]. Bleeding complications increased after paracentesis were high Model for End-stage Liver Disease (MELD) and Child-Pugh scores and renal injury. Low platelet, elevated INR, and low fibrinogen are also important for bleeding after paracentesis [7-9]. It has been shown that evaluation with ultrasound before paracentesis reduces hemorrhagic complications [10,11].

## Conclusions

RSH is more common in patients using anticoagulants or at high risk of bleeding. POCUS allows rapid assessment of patients at high risk in the ED. In addition, the use of ultrasound before paracentesis reduces the complications that may develop. Therefore, it needs to be integrated more into patient care.
